# MyHealthAvatar lifestyle management support for cancer patients

**DOI:** 10.3332/ecancer.2018.849

**Published:** 2018-07-11

**Authors:** Xu Zhang, Zhikun Deng, Farzad Parvinzamir, Feng Dong

**Affiliations:** 1 Centre for Visualisation and Data Analytics, University of Bedfordshire, Luton LU1 3JU, UK; 2 School of Psychology, Queen’s University Belfast, Belfast BT7 1NN, UK

**Keywords:** lifestyle management, app, breast cancer, prostate cancer, mobile

## Abstract

MyHealthAvatar (MHA) is built on the latest information and communications technology with the aim of collecting lifestyle and health data to promote citizen’s wellbeing. According to the collected data, MHA offers visual analytics of lifestyle data, contributes to individualised disease prediction and prevention, and supports healthy lifestyles and independent living. The iManageCancer project aims to empower patients and strengthen self-management in cancer diseases. Therefore, MHA has contributed to the iManageCancer scenario and provides functionality to the iManageCancer platform in terms of its support of lifestyle management of cancer patients by providing them with services to help their cancer management. This paper presents two different MHA-based Android applications for breast and prostate cancer patients. The components in these apps facilitate health and lifestyle data presentation and analysis, including weight control, activity, mood and sleep data collection, promotion of physical exercise after surgery, questionnaires and helpful information. These components can be used cooperatively to achieve flexible visual analysis of spatiotemporal lifestyle and health data and can also help patients discover information about their disease and its management.

## Background

MyHealthAvatar (MHA) [1] is a European Commission funded research project that aims to provide a unique interface that allows data access, collection, sharing and analysis to overcome the shortcomings of existing, highly fragmented resources. It is not only impossible for the patients and doctors themselves to aggregate a large amount of fragmented data collected from different sources but also difficult to make effective analyses of the data. Thus, MHA is designed to be a lifelong companion, providing long-term and consistent health status information of individual patients.

MHA focuses on health self-monitoring, including fitness and lifestyle data. The visualisation components in MHA that facilitate health and lifestyle data presentation and analysis include timelines, clock views, maps and activity graphs; MHA has integrated these features to achieve flexible visualisation of spatial-temporal lifestyle data. MHA provides health data collection, storage and access for patients, doctors and nonmedical users. The data can be automatically collected or manually input [2].

According to National Health Service (NHS) research [3], a healthy lifestyle can give people more control over their health and help them improve it. Many factors can affect health, including body weight, calories and physical activity. There is a strong evidence that weight issues can increase the risks of affecting the treatment of many different types of cancers, including prostate and breast cancers [4, 5]. According to Prostate Cancer UK research, a healthy weight means the cancer is less likely to spread after surgery or radiotherapy [6]; Hormone therapy may also be more effective if patients maintain a healthy weight. Many side effects of treatment are much easier to cope with after surgery while at a healthy weight. Conversely, being underweight is also problematic and affects the health of patients significantly. MHA helps patients by promoting physical activities in order to maintain a healthy weight.

The iManageCancer project aims to empower patients and strengthen self-management in cancer diseases [7, 8]. The MHA platform has been involved in iManageCancer by providing the functionality to support lifestyle management of cancer patients. Moreover, iManagecancer Personal Health Portal (iPHR) can access the MHA data to offer an easy-to-use interface for desktop, tablet and smartphone [9]. Therefore, MHA is a service that targets cancer patients for data collection and self-management services. The system supports the visualisation of behaviours and daily activities of the patients. It functions as a supportive environment to empower patients in self-management, encouraging good lifestyles and behaviours, hence enabling more effective cancer care through improving the patients’ compliance with healthy lifestyle recommendations. It offers a range of tools for the self-management of cancer in terms of lifestyle and physical activities. These tools are mainly designed to target patients with prostate and breast cancers and to help them with the following aspects of their treatment.

### Information collection and access

- Mobile-based global positioning system (GPS) and sensors are used in data collection and information access.
- MHA stores the individual patient avatar data in the iPHR repository where it is accessible to the patient.
- Patient and doctor can check the iPHR data on the MHA apps or iPHR website.

### Data management and sharing

MHA is a tool that allows highly self-motivated data management and user-centred data collection, supported by the necessary data integrity measures. The patients have full rights to share their own data. The individual patient decides how the data is shared with relevant stakeholders. For example, the cancer status data can be shared with physicians for clinical treatment; with research organisations to support clinical research; or with other individual patients on a volunteer basis. These options are supported by the underlying information and communications technology architecture, which has adequate measures to ensure data reliability and integrity.

### Information analysis and visual analysis

The purpose of health and medical information collection is to promote healthy lifestyles of patients and to assist clinical decision making. Information analysis is a vehicle for assisted clinical decision-making and knowledge explorations by which doctors can make basic analyses based on knowledge from the platform, and augment their clinical knowledge with heterogeneous information from the avatar.

### Health questionnaire and exercise

In order to help patients’ recovery and lower the risks after surgery, component apps offer health questionnaires and exercise suggestions for patients. According to the result of the questionnaires, the patients can do a self-check for health status which can inform their consultation with doctors.

### Cancer knowledge

In order to raise the patients’ risk awareness, the MHA app provides the function to improve the patients’ knowledge about health and diseases by tailored information provision.

As the self-management includes health-and-lifestyle data collection, access, analysis, health questionnaire, exercise and cancer knowledge, it is hard for the user to select, view, understand and gain knowledge from a large collection of data without proper visualisation. Thus, visualisation and visual analysis is a critical part of the effective utilisation of data collected and stored on the MHA platform.

This paper focuses on the visual analysis components and is organised as follows. The section ‘*Related work*’ briefly introduces related work on lifestyle collection. The section ‘*Visual analysis components*’ introduces and analyses the visual components in the app. The section ‘*App evaluation and patient feedback*’ presents the app evaluation and patient feedback during the MHA’s pilot study and trial. The last section is the conclusion.

## Related work

With the increasing popularity of fitness and health sensors and apps in recent years, such as Fitbit [10], Withings [11], iHealth [12], Moves [13], etc, there has been increasing interest in building centralised health and fitness data repositories.

Fitbit provides wearable devices which record data such as steps, distance and calories. These devices communicate with a host computer using Bluetooth that sends their data directly to the user’s account on the Fitbit website.

Withings provides devices that measure or calculate data such as steps, distance, calories, heart rate, sleep quality and heart rate. Devices include wristband, watch, scales and blood pressure monitor.

Moves is an app for fitness and lifestyle recording. It automatically records the step number and location of the user and automatically recognises the activity type, such as walking, running, cycling, transport and so on. The user can either view the distance, duration, steps and calories data on the mobile phone or export the data from the Moves server. An automatic daily storyline with time and location are recorded and shown on the map in the Moves app.

Google Map timeline [14] records the user’s location and daily routes and can recognise some of the user’s activities.

HealthVault [15] from Microsoft integrates a number of fitness sensors on the market to provide a central repository and application programming interfaces (APIs) for data access. It enables a connected ecosystem with privacy and security-enhanced foundation including more than 300 applications and more than 80 connected health and fitness devices.

Similar to HealthVaul, MyFitnessCompanion [16] is another healthcare platform for users to manage their personal health data. It includes many metrics such as weight, heart rate variability, blood pressure, food intake, blood glucose, insulin, asthma and so on. Moreover, it has a real-time visualisation mode, which keeps track of and visualises all user measurement with a simple time graph and the ability to share these graphs with others.

These tools can achieve data collection but do not provide powerful visualisation tools for data analysis and health information for users and patients. In addition, the employment of user interactions has not been widely studied in data visualisation and analysis of those applications. In contrast, MHA provides standalone components as well as integrated views for interactive visual analysis of personal health and lifestyle data from multiple heterogeneous data sources.

Healthcare has been an important research and application field of data analysis and visualisation for several decades [17]. Behind healthcare visual analytics, much of the focus is on the visualisation of electronic health records (EHRs). Reference [18] gives a detailed review of the related work, categorising by individual patients or group of patients. Reference [19] also presents a recent review of innovative visual analytics approaches that have been proposed to illustrate EHR data, including [20–25] and so on. Personal health information has been increasingly collectible and accessible in the information era. With the trend of ‘predictive, pre-emptive, personalised and participative’ healthcare [26], more personalised data is desired for predictive analysis of medical care. Valuable lifestyle patterns can be discovered by analysing the personal data collected by sensors and apps. Together with the clinical information that has long played the major role in health and medical decision making, this information can introduce more added value for health monitoring and medical decision making. MHA can not only directly access personal health and lifestyle data from devices such as Fibit, Withings but also analyse the data to extract high-level lifestyle data. Together with appropriate models, MHA aims to provide personalised health monitoring, analysis and risk management based on multiple heterogeneous data sources, which is a key difference from the existing health data sharing platforms or healthcare visual analytics systems.

## Data collection method

MHA data are health and lifestyle oriented and can be categorised into health sensor data and human input data.

### Sensors and apps

In recent years, there has been a boom in the health sensor market. Sensors evolved from traditional devices such as the step meter to Internet-enabled devices such as Fitbit [10], Withings [11] and iHealth [12]. They measure data such as steps, walking distance, calories, sleep quality, heart rate and weight, depending on the device model. Some devices measure more medical oriented data, such as blood pressure and glucose. Meanwhile, with the rise of smartphones, fitness mobile apps are also becoming an important data source for health and lifestyle data. Mobile phones with proper sensors installed are capable of not only measuring the step number with an accelerometer but also recording the location of the user with a GPS sensor, thus keeping track of both the fitness data and daily lifestyle data of the user.

Currently, MHA supports importing data from a user’s Fitbit, Withings and Moves accounts via APIs provided by these devices. More devices and apps will be supported in the future.

### Human input data

In addition to data automatically collected by fitness and lifestyle sensors and apps, there are data that may need manual input such as patient’s daily weight, mood, sleep duration and quality and questionnaire. The app offers the edit panel to allow the patient to input or modify the data. Manual inputting is a very important data source, which can be used not only for data input but also for data editing and error fixing. Consequently, MHA supports human input data in addition to sensor data.

## Visual analysis components

MHA provides several mobile-based components for visualisation and analysis of personal health and lifestyle data, including activity tracking, sitting behaviour monitor, weight-loss program, lifestyle control, health questionnaire, exercise and information.

### Activity tracking

**Figure 1.**
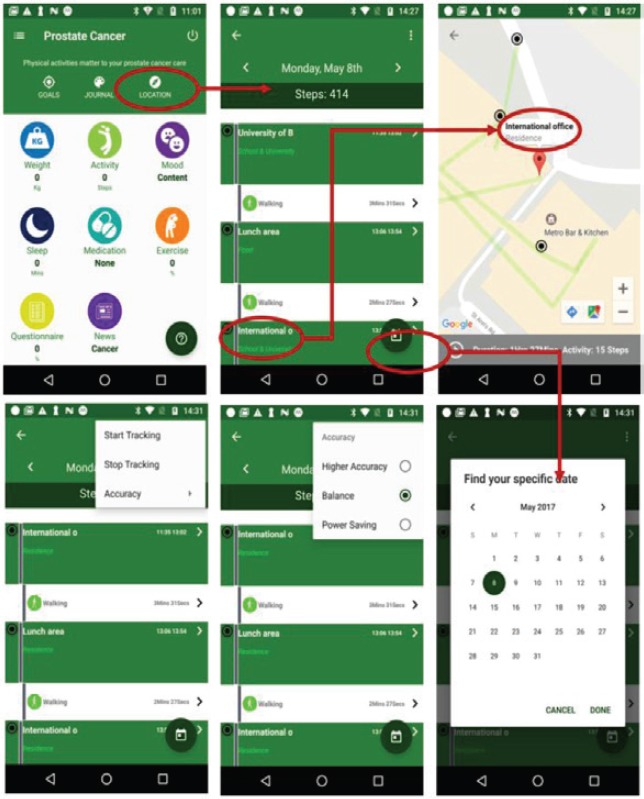
Example screenshot of activity tracking.

As shown in Figure 1, the activity tracking is based on GPS sensors to provide place and activity information across time. It can monitor the calories burnt and also provides information about the patients’ locations and activities. The patients can also navigate backwards through historical or specific records. The use of a map is to make a natural and intuitive spatiotemporal visualisation and analysis of the user’s locations and routes to facilitate analysis, understanding and knowledge discovery of lifestyle. The map implementation is based on Google Maps [27].

### Sitting behaviour monitoring

**Figure 2.**
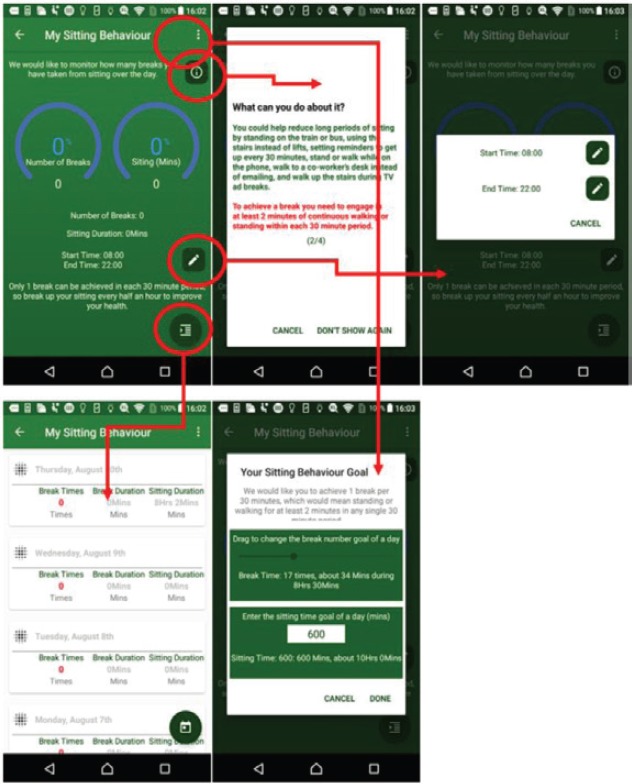
Example screenshot of sitting behaviour monitoring.

Studies have associated excessive sitting with many health problems such as overweight and obesity, and some types of cancer such as prostate and breast cancer [4, 5]. The behaviour monitoring is based on the activity tracking results and mobile sensor to help the patient realise the lengths of sitting and activity time during a day. The patient could choose to reduce long periods of sitting by standing on the train or bus, using the stairs instead of lifts, setting reminders to get up every 30 minutes, stand or walk while on the phone, walk to a co-worker’s desk instead of emailing, and walk up the stairs during TV ad breaks. As shown in Figure 2, patients would receive an alert every 30 minutes, or an interval of their own choosing, to take a break from sitting. Sitting behaviour information is recorded and displayed in a timeline list.

### Weight-loss programme

**Figure 3.**
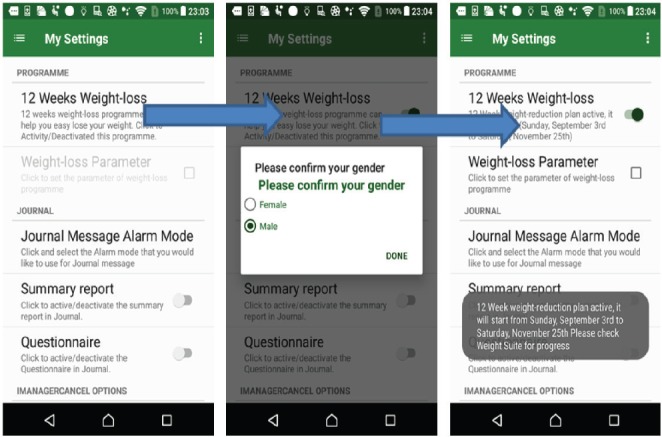
Example screenshot of the weight-loss program.

The 12-week weight-loss programme can help the patients to reduce their weight. Suggestion tips are offered to assist the user’s food and exercise. As shown in Figure 3, this program will update the reduced weight goal week by week. During the programme time period, the program will measure the user’s weight changes and summarise their weight and activities every weekend.

### Lifestyle control

Lifestyle data are inherently time-dependent. To visualise time-varying data, linear and radial layouts can be used. Therefore, the MHA apps provide a timeline layout in the suite functions to allow the management of information on the patient’s weight, activity, sleep and mood state.

**Figure 4.**
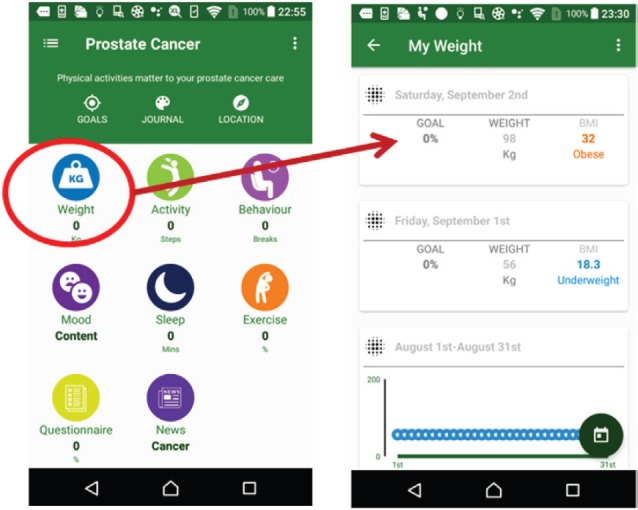
Example screenshot of weight suite.

**Figure 5.**
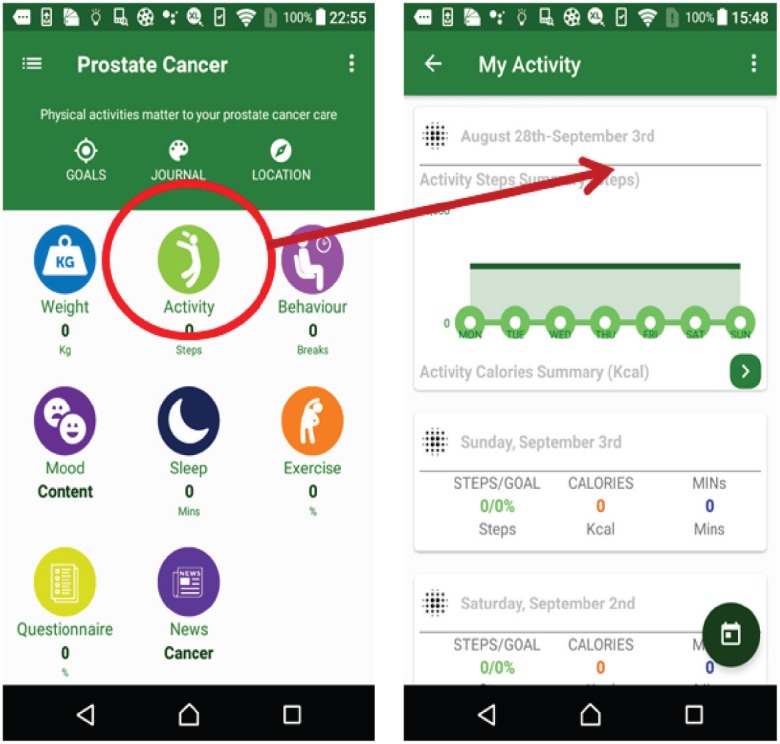
Example screenshot of activity suite.

**Figure 6.**
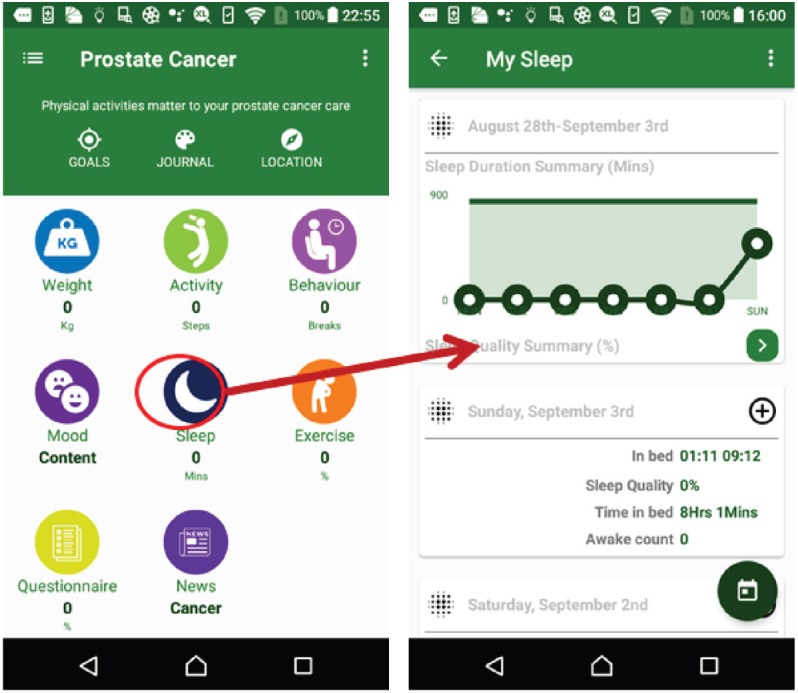
Example screenshot of sleep suite.

**Figure 7.**
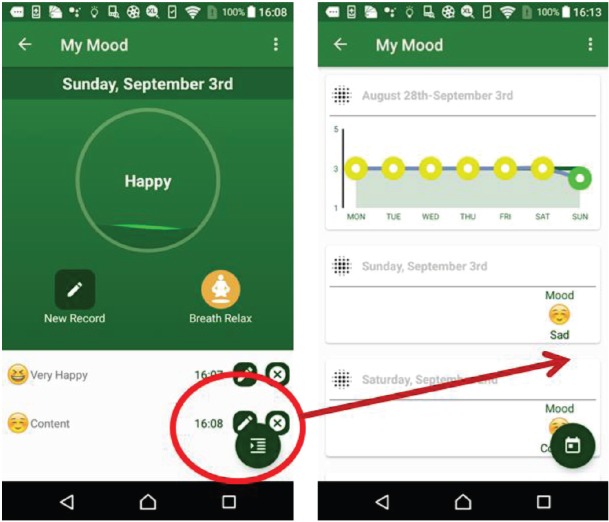
Example screenshot of mood suite.

**Figure 8.**
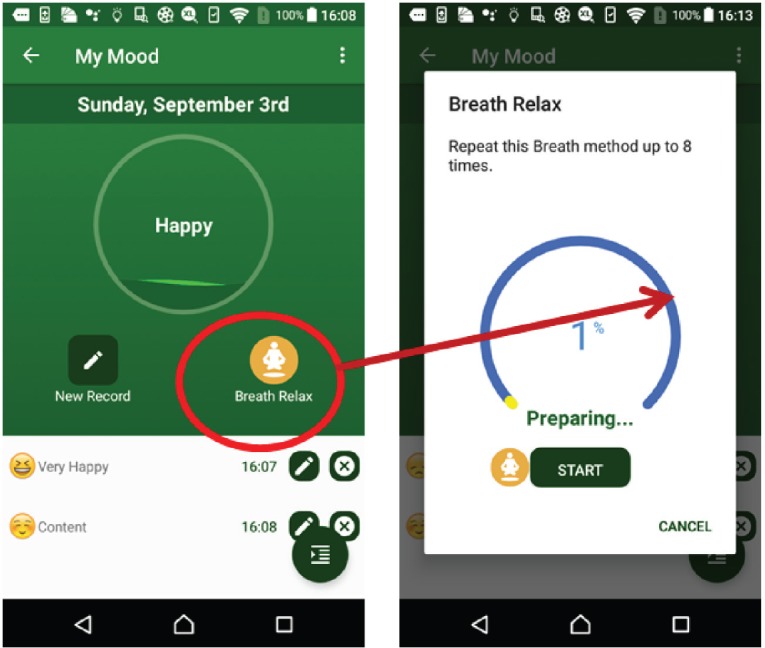
Example screenshot of breath relax program.

- The weight suite manages the patient’s weight information. The patient can access the weight information from Withings or MHA. As shown in Figure 4, this function records the weight and body mass index and the weekly and monthly weight summaries.
- The activity suite manages the patient’s walk steps, distance and duration information. Patients can access the information from Withings, Fitbit, Moves or MHA data source. Patients also can obtain the weekly and monthly activity summaries as shown in Figure 5.
- The sleep suite manages the patient’s sleep information such as duration and quality. Patients can access the sleep information from Fitbit or MHA data source. As shown in Figure 6, patients also can obtain the weekly and monthly sleep summaries on the list.
- The mood suite manages the patient’s mood information. As shown in Figures 7 and 8, patients can not only obtain the records of their mood and weekly/monthly mood summary in the list but also use the relax program to relax in real time.

### Health questionnaire

The health questionnaires monitor the patient’s information after treatment. The MHA apps have implemented different questionnaire components.

### Prostate cancer questionnaire

**Figure 9.**
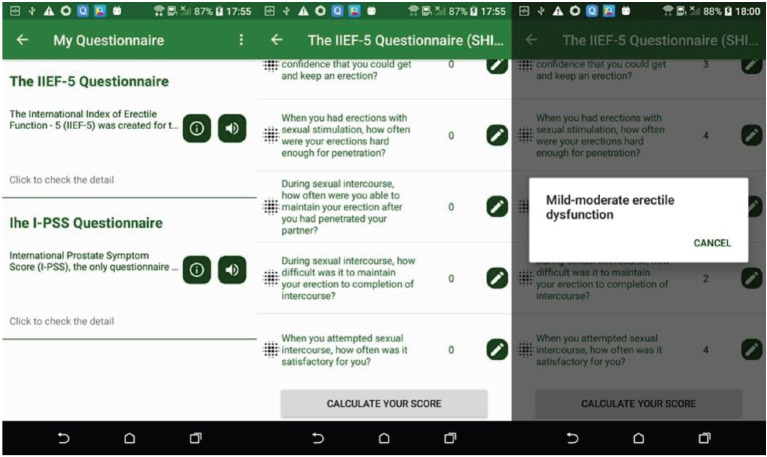
Example screenshot of prostate cancer questionnaire.

In the prostate cancer app, MHA includes the International Index of Erectile Function-5 (IIEF-5) and the International Prostate Symptom Score (I-PSS) questionnaires as shown in Figure 9.

IIEF-5 is a validated, multidimensional and self-administered investigation that has been found useful in the clinical assessment of erectile dysfunction and treatment outcomes in clinical trials [28].

I-PSS is based on the answers to seven questions concerning urinary symptoms and one question concerning quality of life. Each question concerning urinary symptoms allows the patient to choose one out of six answers indicating increasing severity of the particular symptom [29].

### Breast cancer questionnaire

**Figure 10.**
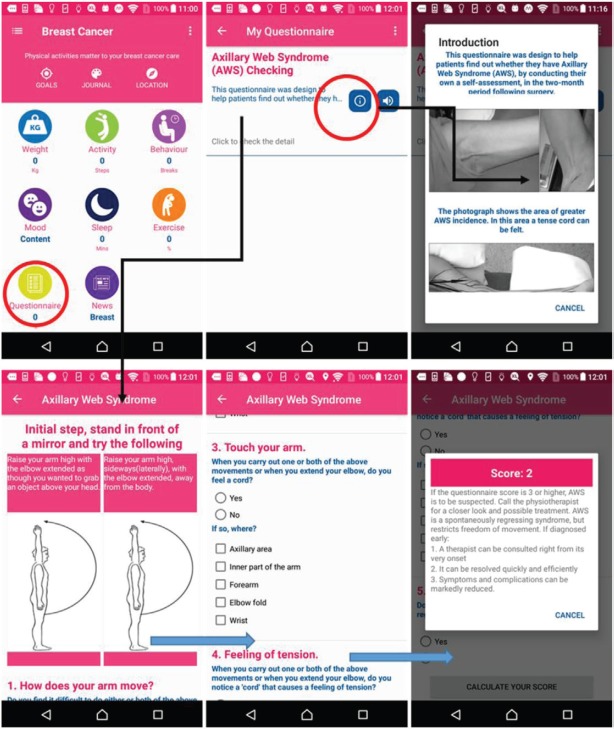
Example screenshot of breast cancer questionnaire.

In the breast cancer app, the MHA questions suite for axillary web syndrome (AWS) after surgery is based on information from the European Institute of Oncology (IEO) hospital, Milan [31]. According to the questionnaire’s result, the apps will give a score to the patient to show the risk level of having AWS as shown in Figure 10.

### Health exercise

Health exercises manage the patient’s exercise information after surgery. MHA is focused on two different cancer diseases, prostate and breast cancer apps have different exercise components.

### Health exercise on prostate cancer

**Figure 11.**
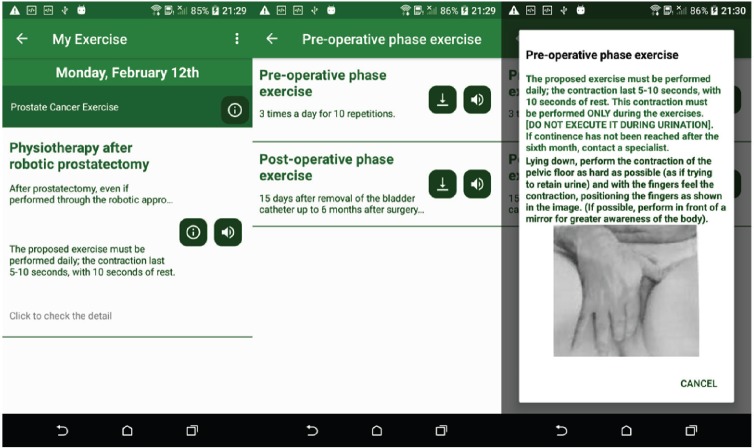
Example screenshot of prostate cancer exercise.

In the prostate cancer app, exercises of contraction and awareness of the pelvic floor have been proposed [31]. After prostatectomy, even if performed through the robotic approach, in some patients, there are cases of urinary incontinence. Performing exercises of contraction and awareness of the pelvic floor can help to reduce urinary leakage and accelerate the process of recovery of continence. The design of the exercise is shown in Figure 11.

### Health exercise on breast cancer

**Figure 12.**
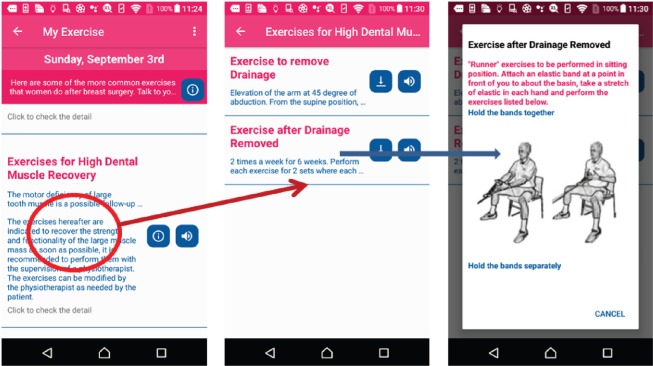
Example screenshot of breast cancer exercise.

In the breast cancer app, MHA provides tailored exercises for breast cancer after surgery, such as exercise after sentinel lymph node biopsy and exercise for high dental muscle recovery. These exercises are proposed by the IEO hospital [31]. Patients customise the exercises for their situation and check their achievement from the recorded list as shown in Figure 12.

### Health information

In order to help patients gain knowledge about their cancer, MHA provides guidance and news for patients with prostate and breast cancer. As mentioned in the sections ‘*Health questionnaire*’ and ‘*Health exercise*’, MHA also offers two different components for different cancer diseases.

### Health information for prostate cancer

**Figure 13.**
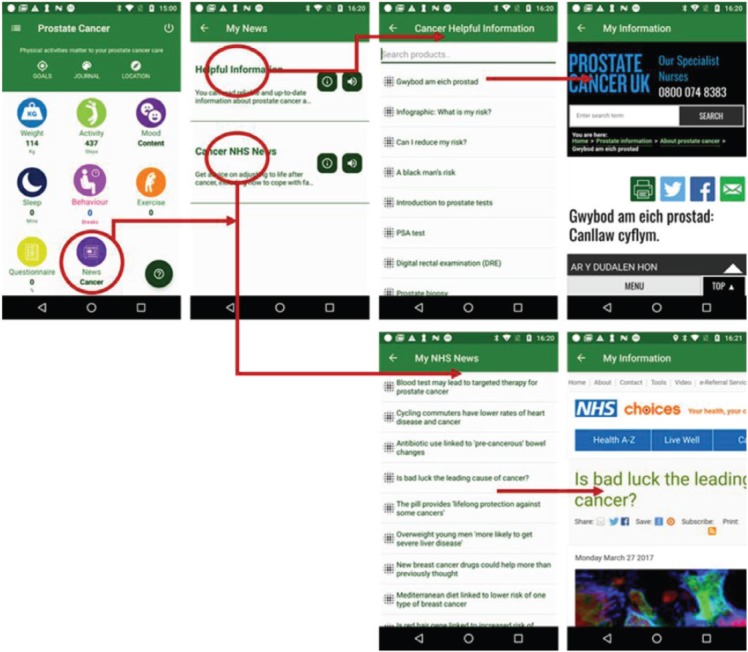
Example screenshot of prostate cancer information.

In the prostate cancer app, MHA provides information by using the resources from Prostate Cancer UK and the NHS, UK. Prostate cancer UK is the national organisation for prostate cancer, in the last 20 years, they have invested over £37 million in groundbreaking research, and continue to provide award-winning support for men [32]. The NHS is the publicly funded national healthcare system for the UK [33]. As shown in Figure 13, by using the filter function, the patients can search and choose items on the list and view the details.

### Health information for breast cancer

**Figure 14.**
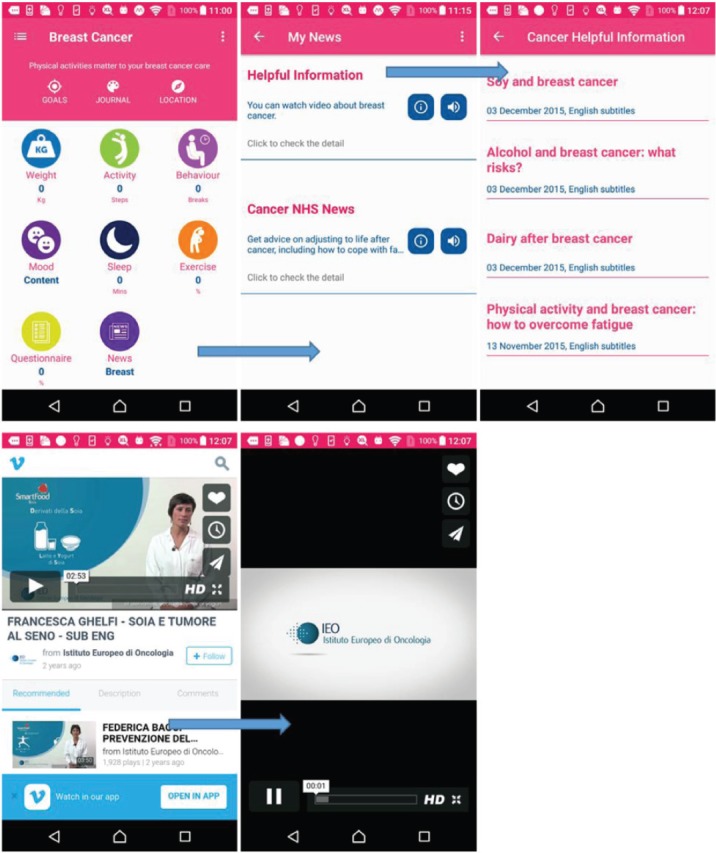
Example screenshot of breast cancer information.

In the breast cancer app, MHA provides information by using the resources from IEO breast cancer information [31] and the NHS. For the NHS, the user can search the topic as mentioned in the section ‘*Health information for prostate cancer*’. As shown in Figure 14, the IEO hospital provides many videos on different topics.

## App evaluation and patient feedback

The apps were tested in three events and feedback from the patients is given in Table 1. The main test parts include account management, data collection and visualisation.

(a) The first workshop involved ten participating cancer survivors and citizens. It took place on 23 January 2017 and then was followed with a second workshop in May 2017 which involved nine patients/citizens [34].

The first workshop allowed and encouraged ten participating cancer survivors and citizens to evaluate both the functionality and design of the apps in the iManageCancer platform. The design of the workshop for testing the app by end users followed the co-discovery methodology.

The ten patients were divided into teams of two. Patients carried out specific pre-determined tasks. They were asked to think out loud and discuss the process with their ‘partners’ whilst navigating through the apps.

Cancer Intelligence/*e*cancer and Tenovus organised remote testing of the platform and services by ten patients in their daily lives for the week 17–24 July 2017, including an interview by phone to provide further information to evaluate the services in practice [34].

As a result of the above-mentioned testing, three common themes emerged:

1. User experience (UX) design
2. Bugs and early development
3. Visual design

End users could see the value and potential of the iManageCancer apps and provided a wealth of constructive feedback. It was agreed that an introductory video to assist patients when using the iManageCancer platform would be developed and integrated as a priority. This is available in English at [35].

Brand and design cohesion would also be addressed during the UX design process; this will involve developing a unified user interface, which will simultaneously improve the user experience and visual design.

There were three main feedback points from the patients:

1. Understanding issue: the patients complain that they don’t know how to use some of the functions in the app.
  Solution: a floating button which can provide the specific introduction to the different function pages has been developed into the app.
2. Internet data usage issue: as many patients’ mobile phones were old and had limited internet data, an issue occurred when they were using the online location tracking function from the moves/fitbit.
  Solution: in order to solve this, a new offline location tracking algorithm has been designed and implemented in the app. This function doesn’t need to sync with the server and can analyse the location information locally.
3. Loading data slow issue: the current data needs quite a long loading time to download to the ‘Listview’ of the app.
  Solution: in order to solve this, a new custom ‘Listview’ has been designed in the app. In the list, instead of loading all data into the app, every page only loads 20 items of the data and patients can load the new data by scrolling down to the bottom of the list. This function improves the data loading slow issue for the patients.

(b) On 18 May 2017, the IEO organised the Patient Empowerment Forum in Milan [34]. At the Forum, two workshops concerning iManageCancer were held. In one of the workshops, a practical demonstration session was performed with about 20 citizens who were not only patients but also healthcare professionals and other participants.

There are three feedback issues arising from the tester:

1. Medication reminder function is duplicated.
  Solution: the medication reminder function has been achieved in another iManageCancer app, so it has been removed from the current app.
2. Breast cancer app issue: due to there being female patients who want the breast cancer app, the breast cancer app has been designed and published in iManageCancer.
3. According to the patients, they require a body status control function. The solution implemented within the apps is the sitting behaviour monitor. It can monitor the patient’s sitting time and also give an alarm if the patient has sat for quite a long time.

(c) In terms of the IEO pilot study [34], we have 138 cancer patients using the app, the groups are:

1. For the breast cancer patients:
  Sixty-eight patients recruited, four drop-outs
  Forty-four patients completed T1 questionnaires
  Forty regular admissions, 19-day surgery, five radiotherapy
2. For the prostate cancer patients:
  Twenty-seven patients recruited, two drop-outs
  Eight patients completed T1 questionnaires
  Twenty-five regular admissions, two radiotherapy

A total of 113 patients refused to participate, so the recruitment rate is 45%. Moreover, the preliminary informal feedback from patients participating in the pilot study and using the platform indicates that: first, they really like the visualisation part of MHA; second, they have many suggestions:

1. Login issue: web-page-based login method has many issues such as delay and compatibility.
  Solution: the new login method which is based on activity has been implemented in the apps.
2. Update the exercise detail for the prostate cancer app based on the IEO hospital’s suggestion.
3. Update the exercise and questionnaire details for the breast cancer app based on the IEO hospital’s suggestion.

All the testing and study events are focused on the user interface and experience, bug-fixing and functionality improvement. According to the feedback from the events, many of the features of the apps have been improved, especially the user experience components, e.g. Internet and power issues. Moreover, IEO will carry out a future study based on the apps; thus the apps will be improved by adding new features based on patient feedback.

## Conclusions

The iManageCancer project provides a cancer disease self-management platform designed according to the specific needs of patient groups. It focuses on the well-being of the cancer patient with special emphasis on psycho-emotional evaluation and self-motivated goals.

MHA for iManageCancer is a solution that offers access, collection and sharing of long-term and consistent personal health status data to iPHR records and also provides an integrated digital representation, which helps prostate and breast cancer patients to:

- Know their health status and performance by viewing their self-collected data
- Improve their knowledge about health and diseases by the tailored information provision
- Raise their risk awareness and perceptions about the diseases by personalised risk assessment
- Engage in health and fitness activities by recommending relevant programmes and courses
- Look after their weight, fitness, calories, emotion and sleep.

In the future, the main work will focus on:

- Fixing the bugs
- Improving the visitation for a better quality of lifestyle review
- To improve the network connection for a better solution for synchronisation with the server
- To reduce the power usage for a better user experience.

## Conflicts of interest

This research is sponsored by the European Union’s Horizon 2020 research and innovation programme under grant agreement No 643529. This paper reflects the authors’ view. The Commission is not responsible for any use that may be made of the information it contains.

All authors declare that there are no conflicts of interest.

## Authors’ contributions

X Zhang designed and implemented the frame and functionality of front end of the proposed MHA apps.

Z Deng designed and implemented the functionality of the back-end of the proposed MHA apps.

Farzad Parvinzamir designed the visualisation of the proposed MHA apps.

Prof F Dong is the leader of the Centre for Visualisation and Data Analytics research group and manages the progress and quality of the MHA app development.

## Figures and Tables

**Table 1.** App evaluation pilot.

Name	Place	Number of Patients	Date
*e*cancer/Tenovus workshops	UK	29	January–July 2017
Patient empowerment forum	Italy	20	May 2017
IEO pilot study	Italy	138	October 2017 to February 2018
